# The Effect of Audible Joint Manipulation Sounds in the Upper Cervical Spine on Brain Wave and Autonomic Nervous System Activity

**DOI:** 10.3390/life15010103

**Published:** 2025-01-15

**Authors:** Dalton Whitman, Rob Sillevis, Matthew Frommelt

**Affiliations:** Department of Rehabilitation Sciences, Florida Gulf Coast University, Fort Myers, FL 33965, USA; dalton.whitman@ufl.edu (D.W.);

**Keywords:** cervical manipulation, audible manipulation sound, electroencephalography, pupillometry, autonomic nervous system

## Abstract

Background: High-velocity, low-amplitude (HVLA) manipulation is a common manual therapy technique used for treating pain and musculoskeletal dysfunction. An audible manipulation sound is commonly experienced by patients who undergo HVLA manipulation; however, there is little known about the effects and clinical relevance of the audible manipulation sound on cortical output and the autonomic nervous system. This study aimed to identify the immediate impact of the audible manipulation sound on brainwave activity and pupil diameter in asymptomatic subjects following an HVLA cervical manipulation. Methods: 40 subjects completed this quasi-experimental repeated measure study design. Subjects were connected to electroencephalography and pupillometry simultaneously, and an HVLA cervical distraction manipulation was performed. The testing environment was controlled to optimize brainwave and pupillometry data acquisition. Pre-manipulation, immediately after manipulation, and post-manipulation data were collected. The presence of an audible manipulation sound was noted. Results: Twenty subjects experienced an audible manipulation sound. Brainwave activity changes were significant (*p* < 0.05) in both the audible manipulation sound and non-manipulation sound groups. Pupil diameter changes (*p* < 0.05) occurred in both eyes of the non-manipulation sound group and in the left eye of the audible-manipulation sound group. Brainwave activity patterns were similar in both groups. Conclusions: The presence of an audible manipulation sound is not required to produce central nervous system changes following an HVLA cervical manipulation; however, the audible manipulation sound does prolong the effects of brainwave activity, indicating a prolonged relaxation effect.

## 1. Introduction

Manual therapy (MT) techniques are commonly utilized to treat musculoskeletal disorders such as low back pain, neck pain, headache, and temporomandibular joint pain [1]. High-velocity, low-amplitude manipulation (HVLA) is one of the most common MT techniques physical therapists utilize [2,3,4]. While the therapeutic mechanisms of HVLA manipulation are still largely unknown, several theories of the mechanical, neurophysiological, and psychological effects have been proposed [5,6,7,8,9,10,11,12,13,14,15]. An audible manipulation sound is commonly experienced by patients who undergo HVLA manipulation; however, its influence on patient outcomes remains unclear [16,17]. Studies have demonstrated that the audible manipulation sound does not predict the success of an HVLA manipulation in asymptomatic subjects [18]. No central nervous system (CNS) benefits from an audible manipulation sound have been demonstrated in subjects with chronic neck pain [19] or subjects with low back pain [20].

Electroencephalography (EEG) is a non-invasive and reliable examination technique that measures the central nervous system through cortical neuronal activity in the brain [21]. This CNS measurement is accomplished with electrodes attached to the scalp that quantify the electrical output of neurons in cortical areas of the brain [21]. Using EEG, real-time brain responses to various visual, tactile, and nociceptive inputs can be observed [22]. There are five brainwave bands that are typically measured using EEG: alpha, beta, gamma, delta, and theta [23]. Alpha waves have a frequency of 8–13 Hz, are predominant during states of relaxation or mental inactivity, and can be depressed by visual or mental attention [24]. Beta waves typically range from 12 to 38 Hz and are associated with increased attention and learning [25]. Gamma waves range from 40 to 100 Hz and are found during states of hyperarousal and conscious attention [25]. Delta waves are high-amplitude, low-frequency waves (0.1–4 Hz) traditionally associated with all stages of sleep in humans [23,26]. Theta waves have a frequency of 4–7 Hz, are present during states of deep relaxation, and are vital for memory formation [23,25]. The consumer-grade Emotiv EEG has been validated as an effective tool for measuring brainwave activity when compared to research-grade EEG systems [27,28].

Another measurable component of the central nervous system is the autonomic nervous system (ANS), which is responsible for involuntary homeostatic body maintenance and is comprised of sympathetic and parasympathetic components [13]. Autonomic dysregulation is associated with pathologies such as chronic pain disorders [10,29,30]. The most common observation with autonomic disorders is a relatively increased sympathetic output or decreased parasympathetic output [13]. A practical method of measuring changes in ANS activity is pupillary dilation (sympathetic) and constriction (parasympathetic) [31]. The use of automated pupillometry as a valid and accurate measurement tool of the ANS response is thoroughly documented [32,33,34].

While joint manipulation targets the intra-articular structures, there is inevitable deformation of peri-articular structures [4]. Some have hypothesized that thoracic manipulation may stimulate the sympathetic ganglia adjacent to the vertebrae and elicit a measurable sympathetic response through observation of an increasing heart rate, rising blood pressure, or pupillary dilation [4,35,36,37]. Others postulate that a cervical manipulation may stimulate the parasympathetic ganglia and subsequently elicit the parasympathetic response, including decreasing heart rate, lowering blood pressure, and pupillary constriction [36,37]. Currently, no studies suggest that sympathetic or parasympathetic responses can be targeted based on the presence of an audible manipulation sound or manipulation location [13,38].

It has been previously demonstrated that joint manipulation has an effect on the sympathetic nervous system (SNS) and subsequent connections to supraspinal mechanisms of the brain [39,40,41]. Ogura et al. [40] demonstrated that an HVLA manipulation performed on neck pain patients affected structures such as the cerebellar vermis, middle temporal gyrus, visual association cortex, inferior prefrontal cortex, and anterior cingulate cortex. Therefore, it has been suggested that SNS changes may be linked to changes in pain modulation by the brain. In addition, a growing body of evidence supports manipulation-induced neural plasticity in various brain structures, such as the cerebellum, basal ganglia, prefrontal cortex, primary sensory cortex, and primary motor cortex [42]. Taylor and Murphy [43] suggested that cervical spine manipulation may alter cortical integration of somatosensory input, which might explain the mechanisms responsible for the pain relief and increased functional ability that were documented after spinal manipulation.

Currently, there are no studies targeting the simultaneous interactions of the brain and ANS following HVLA cervical manipulation. Moreover, no known association exists between the audible manipulation sound and changes in cortical output or ANS response following such manipulation. Alterations in cortical activity and ANS response may lead to a greater understanding of the neurophysiologic mechanisms underlying HVLA manipulation. If these changes are observed more significantly in the audible manipulation sound group, it might suggest that the audible manipulation sound provides an increased potency of the neurophysiological effects associated with HLVA manipulation. Therefore, this study aimed to investigate the immediate impact of the audible manipulation sound on brainwave activity and pupil diameter in asymptomatic subjects.

## 2. Materials and Methods

This quasi-experimental study used a method of convenience sampling with a within-subject repeated measure design. Institutional review board (IRB# 2020-64, August 2021) approval was obtained from Florida Gulf Coast University (FGCU). This study was also registered at https://clinicaltrials.gov/ with ID# NCT04542707. Subjects were recruited from the student body of the Marieb College of Health and Human Services at FGCU via email and verbal correspondence. All subjects provided written consent prior to participating in the study. Subjects were recruited over three months. The sample size was determined from a previous study using a power analysis with an effect size of 0.20, a significance of 0.05, and a power of 0.60 [44]. It was determined that 36 individuals were required, and ultimately, 40 individuals participated in the study.

A total of 40 asymptomatic healthy subjects were recruited. Subjects were screened for the eligibility criteria. The subjects had to be between 18 and 65 years old, have no history of cervical spine injury within six months of the experiment, and have no history of concussion, traumatic brain injury (TBI), or brain damage to be included in this study. Exclusion criteria were subjects with pain, being outside the age range, presence of contraindications for cervical manipulation including vertebral artery insufficiency and upper cervical instability, conditions or medications that could lead to skeletal compromise, active headache or other pain-inducing disorders, previous traumatic brain injury, and any condition that alters the function of the ANS.

### 2.1. Study Protocol

**Figure 1:**
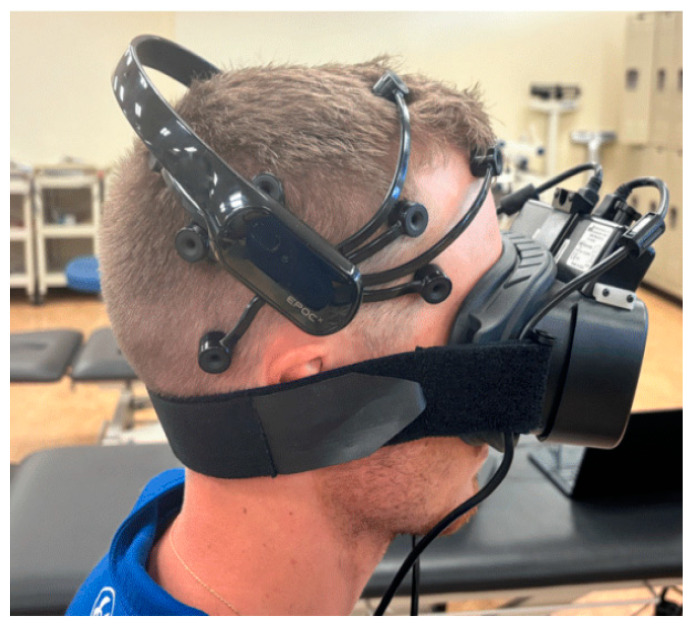
Subjects seated with both the Emotiv EPOC+ electroencephalography and Micromedical Vorteq pupillometer.

All testing was performed in the same isolated room where sound, temperature, and light were controlled and remained constant with minimal electrical interference. The light was dimmed during testing to prevent light from influencing the pupillometry data. This controlled environment should have optimized the acquisition of EEG and pupillometry data. Each subject was seated, and the Emotiv EPOC+ EEG (Emotiv, San Francisco, CA, USA) was placed using the international 10–20 system of electrode placement (Figure 1) [45]. A saline solution was used on each EEG electrode to ensure proper connectivity. Following this, the Micromedical pupillometer (Micromedical Technologies, Inc., Chatham, IL, USA) was placed with the strap around the lowest part of the occiput to ensure that it would not interfere with the EEG electrodes (Figure 1). The device created a completely dark environment for the subject. This was confirmed by subjects prior to data collection. This dark environment maximizes the ability to capture the true autonomic response to the intervention. The validity and reliability of the Emotiv EPOPC+ and the Micromedical pupillometry devices have previously been demonstrated [19,27,28,32,33,34].

Once all equipment was secure, subjects were placed in a supine pre-intervention position with the practitioner’s hands supporting their head. Subjects were instructed to remain relaxed while the practitioner controlled their head and neck position to deliver the intervention. Subjects were informed that a “quick pull” would be delivered to the upper cervical spine, but they were not informed when the manipulation would take place once they were in the pre-intervention position. Additionally, subjects were instructed to keep their eyes open to the best of their ability for the duration of the intervention to capture the pupil responses maximally. Proper connectivity of each device was observed, and a different researcher dimmed the lights of the treatment room.

**Figure 2:**
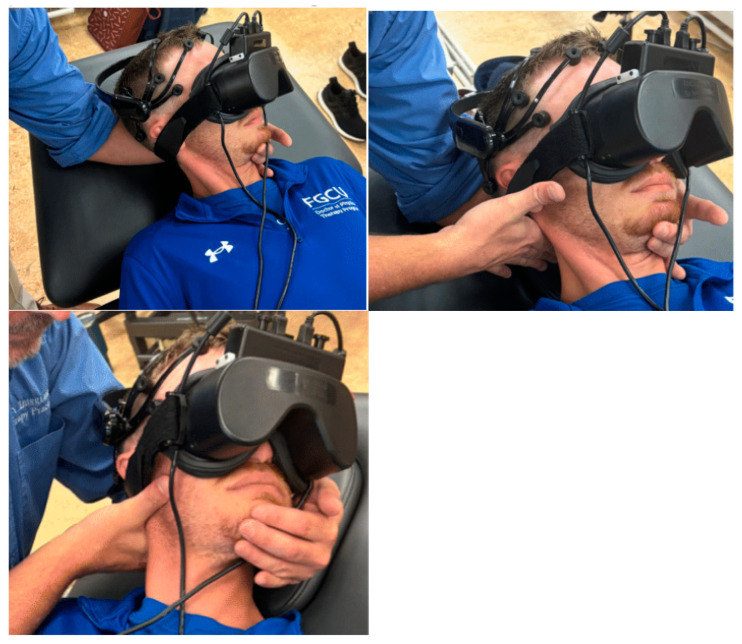
Setup for the atlantoaxial long-axis thrust manipulation with the subject wearing the EEG and pupillometry device. **Upper left**: The practitioner positions the subject in a left rotation and right side-bend with a chin tuck. **Upper right**: The practitioner places the first metacarpophalangeal joint of the manipulating hand on the transverse process of the C1 vertebrate. **Lower**: Atlantoaxial long-axis thrust manipulation pre-intervention position. A high-velocity, low-amplitude thrust manipulation was performed with a direction of force parallel to the practitioner manipulating the forearm, as seen in the lower image.

All data collection measures lasted for one continuous minute. One minute was chosen to make sure that any delayed CNS responses could still be captured. The collection began when both the participant and the practitioner were in the pre-intervention position to ensure that the baseline measurement included static physical touch. To maintain consistency of the tactile experience for each subject, their head was maintained in the manipulation position following the intervention for the entire data collection period. A Fellow in the American Academy of Orthopedic Manual Physical Therapy with 30 years of experience performed the participant’s cervical distraction thrust manipulation. A long-axis distraction manipulation (Figure 2) of the atlantoaxial joint was chosen for this study to limit possible vertebral artery compromise.

Only one manipulation was allowed per subject to isolate nervous system changes to this singular event. Each subject received the atlantoaxial joint long-axis HVLT manipulation on the right side, allowing for consistent and meaningful brainwave and autonomic comparison. Data for both devices were collected at three distinct points: a 10 s average of the subjects’ pre-manipulation (Pre-M) baseline, a 5 s average immediately after the manipulation (IA-M), and a 40 s average post-manipulation (Post-M). After the manipulation, a longer Post-M average was used to maximize the period of observable cortical and autonomic changes.

### 2.2. Statistical Analysis

The Friedman test was used to identify changes in averages of the five brainwave frequency types and pupil diameter that occurred throughout the entire minute of data collection. When a significant finding was identified, the Wilcoxon test was used to determine if significant changes were present between either pre-manipulation (Pre-M) to immediately after manipulation (IA-M), IA-M to post-manipulation (Post-M), and Pre-M to Post-M.

## 3. Results

All 40 subjects completed the study. The study group consisted of 22 males and 18 females, with a mean age of 26.4. Based on the presence of an audible joint manipulation sound during the atlantoaxial joint HVLA manipulation, the subjects were placed either in the audible sound (AS) group or the non-manipulation sound (NAS) group for statistical analysis. Both groups consisted of 20 subjects. Neither group reported any adverse events (including soreness or pain) following the intervention. Statistical analyses were performed using IBM’s SPSS, version 28.0, statistical software package. All data were analyzed using a significance level of 0.05 and a 95% confidence interval. The Shapiro–Wilk test of normality was performed to determine if a normal distribution was present for each frequency band assessed (alpha, beta H, beta L, gamma, and theta waves) and for pupil diameter. The test revealed that the data were not normally distributed (*p* < 0.05); therefore, parametric statistics criteria were not met.

### 3.1. Electroencephalography Analysis

**Figure 3:**
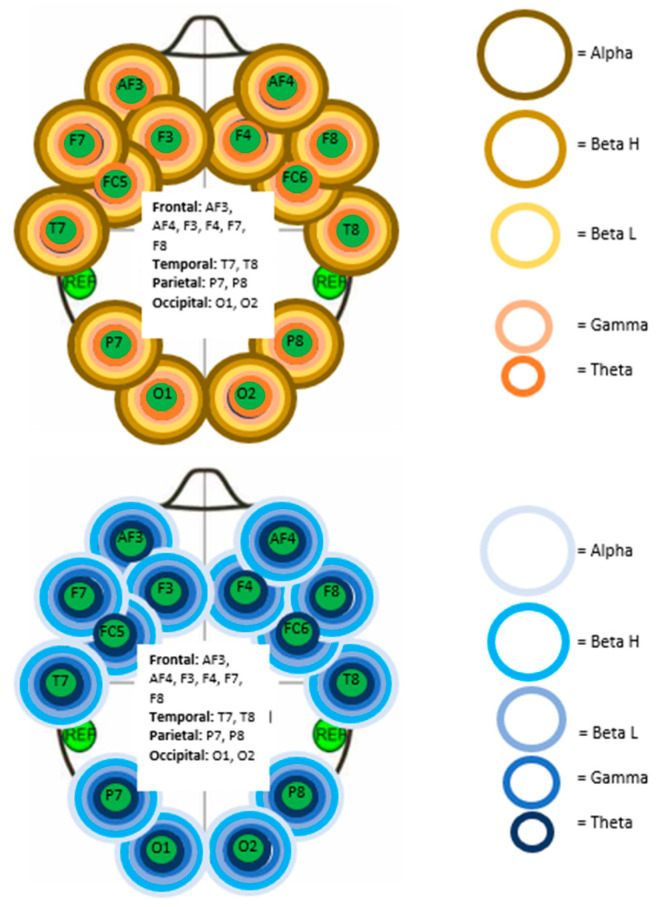
Electrode name and location for electroencephalography. Visualization of significant (*p* < 0.05) Friedman test findings for electroencephalography of NAS (**above**) and AS group (**below**). Ring colors correspond to brainwave bands, as shown on the right.

The Friedman test determined that significant changes (*p* < 0.01) were present in the AS and NAS groups for each of the five brainwave frequency bands (alpha, beta H, beta L, gamma, and theta) at all 14 electrodes of the Emotiv EEG device (5 frequency bands × 14 electrodes = 70 total brainwaves measured). Electrodes were placed based on the brain region (Figure 3) according to the international 10–20 system [45]. Figure 3 displays the visualization of significant brainwave activity changes in both the AS and NAS groups.

The Wilcoxon test identified significant changes (*p* < 0.05) in brainwave activity between each data collection period for the AS and NAS groups. The NAS group displayed an initial (Pre-M to IA-M) decrease in brainwave activity (*p* < 0.05) in each of the five brainwave frequency bands at all 14 electrodes. This decrease in activity was immediately followed by a significant (*p* < 0.05) increase in brainwave activity (IA-M to Post-M). However, the NAS group had no significant change between the Pre-M and Post-M measures.

The Wilcoxon test identified a similar trend in the AS group. An initial (Pre-M to IA-M) significant decrease (*p* < 0.05) in brainwave activity was present in all brainwaves measured. This was followed by a significant increase (*p* < 0.05) in brainwave activity in all but five (93%) of the brainwaves that were measured. The five brainwave types and their locations that did not increase significantly from IA-M to Post-M can be found in Table 1.

A significant (*p* < 0.05) decrease in brainwave activity was observed in 59 of the 70 brainwaves measured (84%) when comparing Pre-M to Post-M activity. Table 2 shows the brainwave types that were not found to be significant during this time interval.

### 3.2. Pupillometry Analysis

The Friedman test identified significant changes in pupil diameter (*p* < 0.05) in the AS and NAS groups (Table 3). The NAS group experienced a significant change in pupil diameter in both eyes, while the AS group had a significant change in the left eye only.

Pupil diameter changes were further analyzed using a Wilcoxon test to determine if there was a significant change between measures in each group (Table 4). The Wilcoxon identified one area of significance (*p* < 0.05) in the left eye of the AS group only. Significant (*p* = 0.021) pupil dilation (sympathetic response) occurred when comparing the IA-M to Post-M data of the AS group. No significant changes were found in other data periods for either group.

## 4. Discussion

This study aimed to identify if the presence of an audible joint manipulation sound resulted in different responses in cortical and autonomic activity following an upper cervical long-axis distraction manipulation targeting the right atlantoaxial joint. Additionally, this study provides a protocolized approach for measuring cortical and ANS changes using electroencephalography and pupillometry for future studies, including symptomatic individuals. Our results showed similar trends in EEG activity among AS and NAS groups. The Friedman test showed that each group experienced identical statistically significant changes (*p* < 0.05) in brainwave activity across all frequency bands under all electrodes. However, the Wilcoxon test identified one major difference in the trend of brainwave activity between the AS and NAS groups.

The brainwave activity of the NAS group was characterized by an initial (Pre-M to IA-M) significant decrease (*p* < 0.05), followed by a significant increase (IA-M to Post-M) (*p* < 0.05). However, no significant change was found when comparing Pre-M to Post-M brainwave activity in the NAS group (*p >* 0.05). This absence of change from Pre-M to Post-M was observed in all brainwave frequencies at all electrodes, and it indicates that the gross brainwave activity of the NAS group returned to baseline levels within 40 s following the manipulation.

Brainwave activity of the AS group showed a similar response pattern of an initial decrease followed by a relative increase in brainwave activity. However, there remained a significant decrease (*p* < 0.05) in cortical activity when comparing Pre-M to Post-M in most brainwave bands (84%) under all electrodes, which seems to indicate that the AS group experienced a greater depression in brainwave activity compared to the NAS group, which might indicate a prolonged relaxation effect. Brainwave frequencies that were not decreased from Pre-M to Post-M in the AS group were widespread and included gamma, beta, and theta waves in the left occipital lobe, left parietal lobe, right frontal lobe, right temporal lobe, right parietal lobe, and right occipital lobe (Table 4). The absence of significant changes (*p* > 0.05) in these waveforms suggests that brainwave activity in these locations either remained neutral or the change was too short to be captured. Our overall findings suggest that the AS may produce a global depression in cortical activity; however, the clinical relevance of this finding remains unknown.

Another unique marker of the AS group was that certain brainwave frequencies from IA-M to Post-M were not found to be significant (*p* > 0.05) (Table 3). These frequencies were gamma waves in electrodes located in the right frontal and parietal lobes. While these gamma waves underwent a significant decrease (*p* < 0.05) from Pre-M to IA-M, they did not return to baseline in the subsequent measure (IA-M to Post-M). Conversely, the IA-M and Post-M measurements showed an increase in alpha, beta, and theta waves. The increase in alpha and theta waves supports the theme of a relaxed state in the brain following the intervention, as alpha and theta waves predominate in states of relaxation and mental inactivity [23,24]. While beta waves are traditionally associated with states of attention [25], Ossebaard [46] has shown that stimulation of beta waves was effective in reducing emotional exhaustion and anxiety. Gamma waves are primarily found during states of alertness or conscious attention [25], so their absence indicates that a relaxation or inhibitory state was further induced ipsilateral to the treatment side in the right frontal and parietal lobes of the AS group. Future studies correlating EEG data and subjective participant experiences in relation to HVLA manipulation are required to support our hypothesis.

The differences in pupillometric data between AS and NAS groups were less defined. By placing each subject in a dim environment, we aimed to limit the influence of light exposure and visual fixation on the pupillary light reflex [47] in an effort to reflect the association between cortical output and autonomic fluctuations accurately. While the statistical analysis revealed pupillometric changes in the left and right eyes for both groups, there is limited evidence to suggest that the intervention was directly responsible for these changes. The Wilcoxon test revealed only one significant instance (*p* < 0.05) of pupil dilation in the left eye from IA-M to Post-M of the AS group. This instance of pupil dilation was accompanied by the previously discussed increase in brainwave activity. While specific brainwave types are not directly responsible for pupillary changes, it has been documented that pupillary dilation can occur as a consequence of a sudden event or unexpected stimuli [47,48]. The subjects were informed that the manipulation would occur, but they could not anticipate the exact moment when the intervention was performed. It is unclear whether the increase in cortical activity indirectly stimulated pupil dilation or if the sudden nature of the manipulation created a sympathetic response. Another consideration for this response is that the right-sided manipulation could have stimulated a strong left-sided central nervous system response via the anterolateral spinothalamic system [49]. Auditory response of the right side was also considered, but there is no evidence in the EEG data (for instance, temporal lobe activity differences between groups) to support this claim. Pupil diameter may fluctuate based on absolute or relative changes in the sympathetic and parasympathetic nervous systems, which, in this case, suggests that an absolute increase in sympathetic activity or a relative decrease in parasympathetic activity was responsible for the observed pupil dilation [50,51,52,53,54]. There were no other instances of pupil diameter changes found between measurement groups, potentially indicating that the significant findings from the Friedman test could be due to normal pupil fluctuations and not attributable to the intervention in either group. These results are consistent with other studies demonstrating that autonomic nervous system activity does not correlate to the audible joint manipulation sound [4,19].

### Limitations

The Emotiv EPOC+ EEG device used does not capture delta brainwave activity. Therefore, no impression was obtained regarding any changes in delta waves. Since delta waves are predominantly associated with sleep in humans, this should not have impacted the outcomes. Only one study identified delta waves being present in awake adults [26]. Adding delta wave measurements in a future study would provide insight into manipulation effects on low-frequency brainwave activity typically more associated with relaxation. Additionally, a placebo effect has been documented regarding the presence of an audible manipulation sound for patients with pain, especially if the subject believes that an audible manipulation sound will lead to a better chance of improvement [4,12,14,15]. While the subjects in this study were asymptomatic, they were not screened for any previous experience with cervical HVLA manipulation, and it is possible that their previous experiences or expectations influenced their central nervous system response. Maintaining a reliable connection to the EEG headset was challenging due to the position required for the cervical manipulation. Although 100% connectivity was achieved before the manipulation, it is difficult to assess how well the connection was sustained during the manipulation. Pupillometry may be affected by the device not focusing appropriately on the pupil in subjects that wore mascara or other dark-colored eye makeup. However, the sample was large enough for this not to impact the findings. Although the location of the data collection was chosen to limit any factors that could influence brainwave and pupil activity, there was an occasional noise factor that could not be controlled. The small study size and emphasis on asymptomatic individuals limit the generalizability of this study. Future studies should include symptomatic subjects in which manipulation is indicated, which would strengthen the findings of this study. Additionally, future studies should determine if a mock manipulation with a simulated audible manipulation sound will yield findings similar to those demonstrated in the AS group.

## 5. Conclusions

A consistent trend was established with EEG findings suggesting that manipulation induces an initial depression of brainwave activity followed by a relative increase in cortical activity, regardless of the presence of the audible joint manipulation sound. However, the audible manipulation sound does seem to prolong the effects of brainwave activity depression, indicating a state of relaxation, although the return to baseline timeframe is unknown. The clinical applicability of the audible manipulation sound in regulating the ANS remains unknown. The clinical relevance of the current findings requires future studies to explore the duration of this depressed brainwave activity and to compare responses between asymptomatic and symptomatic groups.

## Figures and Tables

**Table 1:** Brainwave bands and locations were not found to be significant (*p* > 0.05) when comparing activity from immediately after manipulation to post-manipulation in the audible sound group.

Right Frontal	Right Parietal
AF4 gamma	P8 gamma
F4 gamma	
F8 gamma	
FC6 gamma	

**Table 2:** Brainwave bands and locations were not found to be significant (*p* > 0.05) when comparing activity from pre-manipulation to post-manipulation. AS = audible sound.

Left Parietal	Left Occipital	Right Frontal	Right Temporal	Right Parietal	Right Occipital
P7 gamma	O1 beta O1 gamma	F4 gamma F4 theta F8 theta	T8 beta H T8 gamma T8 theta	P8 gamma	O2 gamma

**Table 3:** Friedman analysis of variance for AS and NAS groups regarding pupil diameter changes. *P* = power.

	AS Left Eye	AS Right Eye	NAS Left Eye	NAS Right Eye
N	20	20	20	20
Chi-Square	6.700	6.100	7.600	5.700
Degrees of Freedom	2	2	2	2
*p*-value	0.035	0.047	0.022	0.058

**Table 4:** Wilcoxon signed a rank test for both groups regarding pupil diameter changes. Pupillary measurements were compared between these timeframes: pre-manipulation (Pre-M) to immediately after manipulation (IA-M), IA-M to post-manipulation (Post-M), and Pre-M to Post-M. Z values followed by “n” indicate a relative decrease in pupil diameter. Z values followed by “p” indicate a relative increase in pupil diameter. *P* = power, NAS = no audible manipulation sound, AS = 0 audible manipulation sound.

	Group	Left Pre-M to IA-M	Left IA-M to Post-M	Left Pre-M to Post-M	Right Pre-M to IA-M	Right IA-M to Post-M	Right Pre-M to Post-M
Z	NAS	−1.643n	−0.859n	−0.149n	−1.829n	−0.597n	−1.904p
*p*-value	NAS	0.100	0.391	0.881	0.067	0.550	0.057
Z	AS	−0.971n	−2.315p	−0.523n	−1.419n	−0.709p	−0.821n
*p*-value	AS	0.332	0.021	0.601	0.156	0.478	0.411

## Data Availability

The original contributions presented in the study are included in the article; further inquiries can be directed to the corresponding author.
